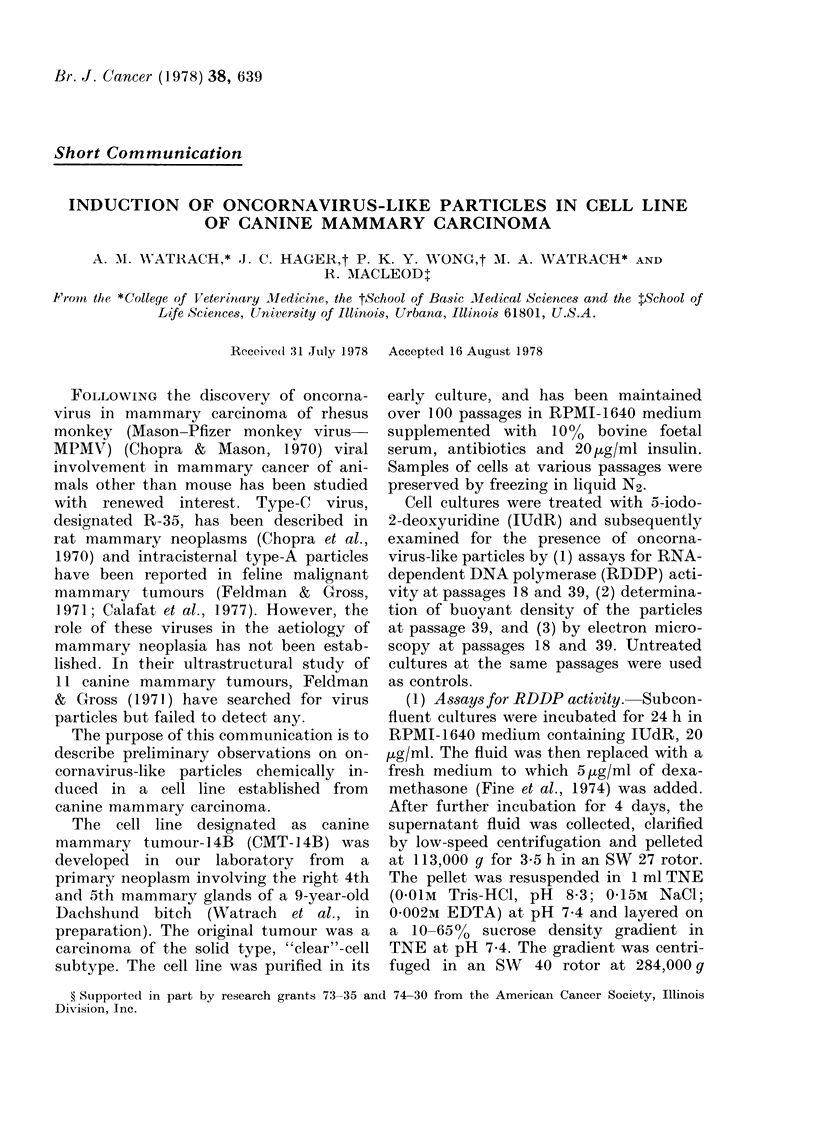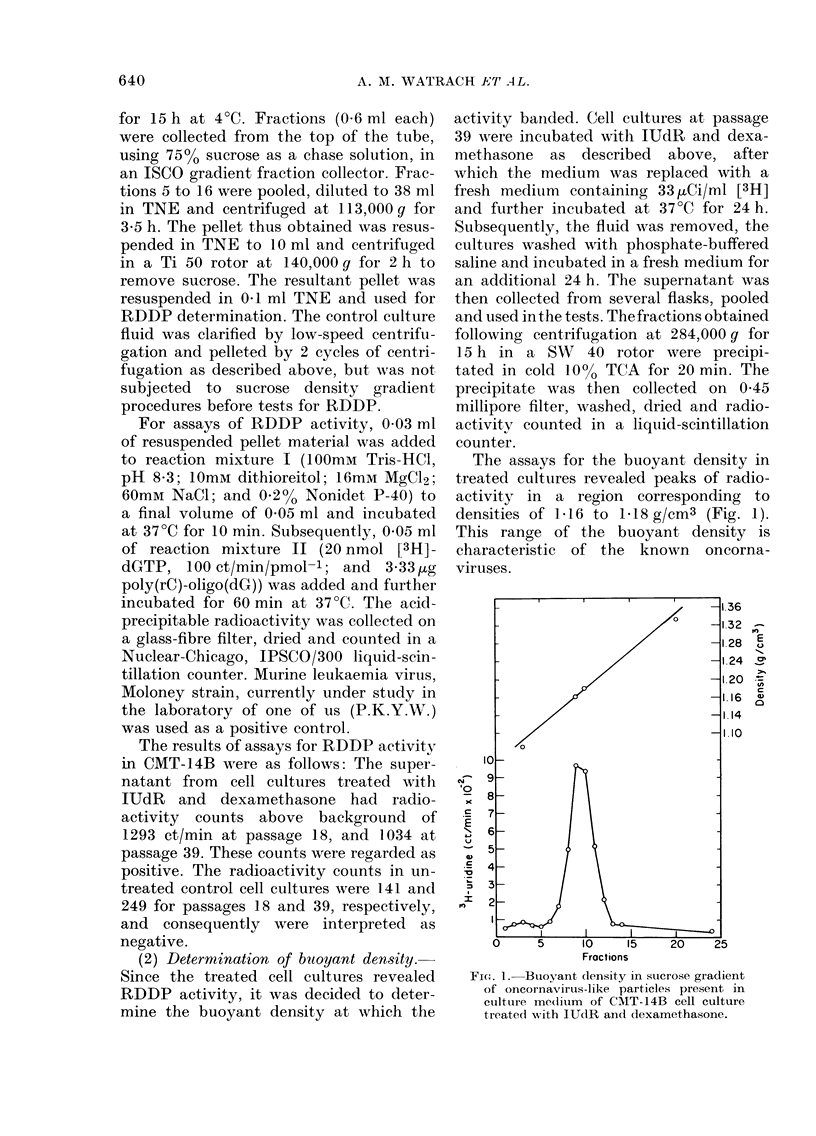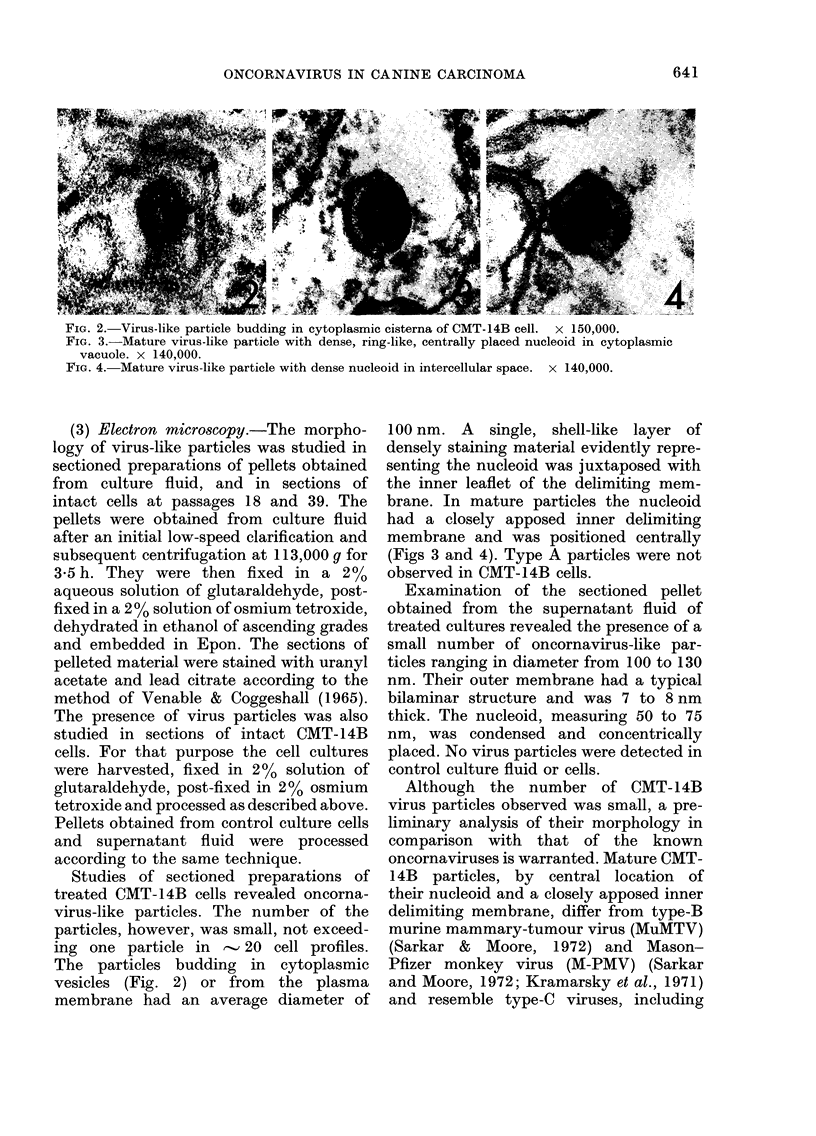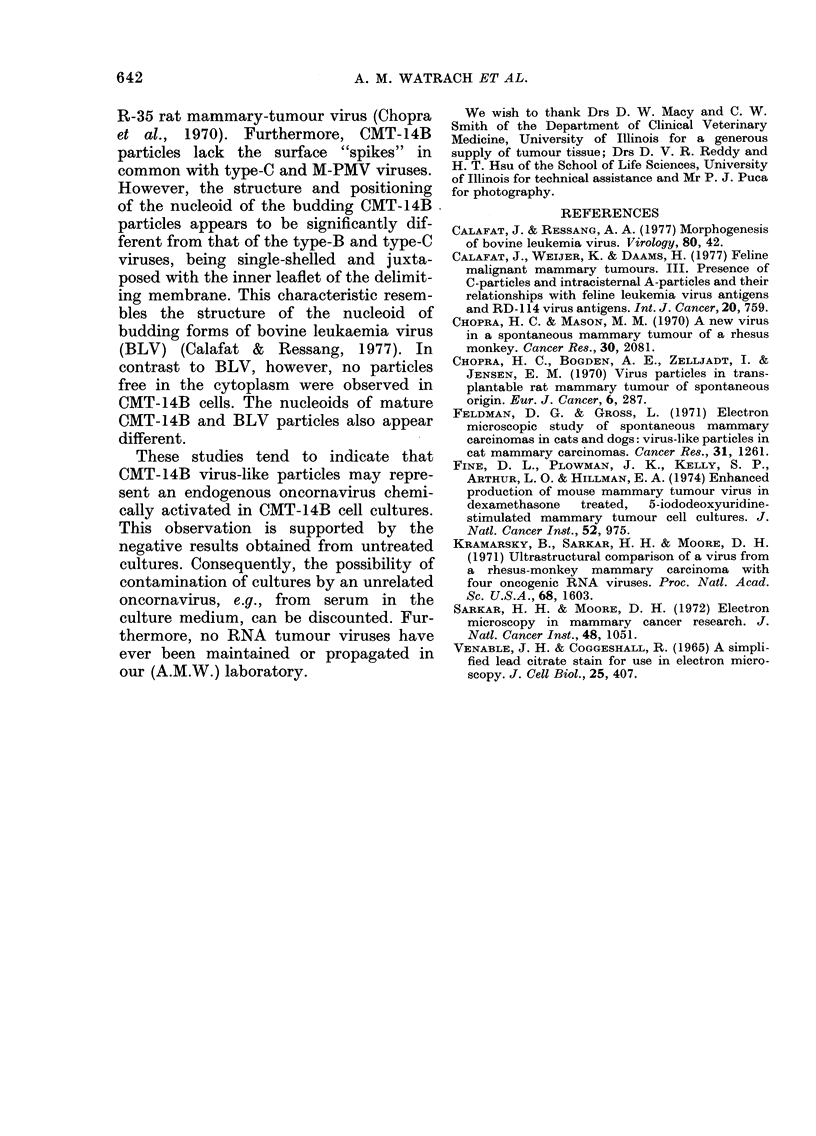# Induction of oncornavirus-like particles in cell line of canine mammary carcinoma.

**DOI:** 10.1038/bjc.1978.263

**Published:** 1978-11

**Authors:** A. M. Watrach, J. C. Hager, P. K. Wong, M. A. Watrach, R. MacLeod

## Abstract

**Images:**


*Br. J. Cancer* (1978) **38**, 639

### *Short Communication*

### INDUCTION OF ONCORNAVIRUS-LIKE PARTICLES IN CELL LINE OF CANINE MAMMARY CARCINOMA

A. M. WATRACH,* J. C. HAGER,† P. K. Y. WONG,† M. A. WATRACH* AND R. MACLEOD‡

*From the *College of Veterinary Medicine, the †School of Basic Medical Sciences and the ‡School of Life Sciences, University of Illinois, Urbana, Illinois* 61801, *U.S.A.*

Received 31 July 1978 Accepted 16 August 1978

FOLLOWING the discovery of oncornavirus in mammary carcinoma of rhesus monkey (Mason–Pfizer monkey virus—MPMV) (Chopra & Mason, 1970) viral involvement in mammary cancer of animals other than mouse has been studied with renewed interest. Type-C virus, designated R-35, has been described in rat mammary neoplasms (Chopra *et al.*, 1970) and intracisternal type-A particles have been reported in feline malignant mammary tumours (Feldman & Gross, 1971; Calafat *et al.*, 1977). However, the role of these viruses in the aetiology of mammary neoplasia has not been established. In their ultrastructural study of 11 canine mammary tumours, Feldman & Gross (1971) have searched for virus particles but failed to detect any.

The purpose of this communication is to describe preliminary observations on oncornavirus-like particles chemically induced in a cell line established from canine mammary carcinoma.

The cell line designated as canine mammary tumour-14B (CMT-14B) was developed in our laboratory from a primary neoplasm involving the right 4th and 5th mammary glands of a 9-year-old Dachshund bitch (Watrach *et al.*, in preparation). The original tumour was a carcinoma of the solid type, "clear"-cell subtype. The cell line was purified in its early culture, and has been maintained over 100 passages in RPMI-1640 medium supplemented with 10% bovine foetal serum, antibiotics and 20 μg/ml insulin. Samples of cells at various passages were preserved by freezing in liquid $N_2$.

Cell cultures were treated with 5-iodo-2-deoxyuridine (IUdR) and subsequently examined for the presence of oncornavirus-like particles by (1) assays for RNA-dependent DNA polymerase (RDDP) activity at passages 18 and 39, (2) determination of buoyant density of the particles at passage 39, and (3) by electron microscopy at passages 18 and 39. Untreated cultures at the same passages were used as controls.

(1) *Assays for RDDP activity.*—Subconfluent cultures were incubated for 24 h in RPMI-1640 medium containing IUdR, 20 μg/ml. The fluid was then replaced with a fresh medium to which 5 μg/ml of dexamethasone (Fine *et al.*, 1974) was added. After further incubation for 4 days, the supernatant fluid was collected, clarified by low-speed centrifugation and pelleted at 113,000 *g* for 3·5 h in an SW 27 rotor. The pellet was resuspended in 1 ml TNE (0·01M Tris-HCl, pH 8·3; 0·15M NaCl; 0·002M EDTA) at pH 7·4 and layered on a 10–65% sucrose density gradient in TNE at pH 7·4. The gradient was centrifuged in an SW 40 rotor at 284,000 *g*

§ Supported in part by research grants 73–35 and 74–30 from the American Cancer Society, Illinois Division, Inc.

for 15 h at 4°C. Fractions (0·6 ml each) were collected from the top of the tube, using 75% sucrose as a chase solution, in an ISCO gradient fraction collector. Fractions 5 to 16 were pooled, diluted to 38 ml in TNE and centrifuged at 113,000 *g* for 3·5 h. The pellet thus obtained was resuspended in TNE to 10 ml and centrifuged in a Ti 50 rotor at 140,000 *g* for 2 h to remove sucrose. The resultant pellet was resuspended in 0·1 ml TNE and used for RDDP determination. The control culture fluid was clarified by low-speed centrifugation and pelleted by 2 cycles of centrifugation as described above, but was not subjected to sucrose density gradient procedures before tests for RDDP.

For assays of RDDP activity, 0·03 ml of resuspended pellet material was added to reaction mixture I (100mM Tris-HCl, pH 8·3; 10mM dithioreitol; 16mM $MgCl_2$; 60mM NaCl; and 0·2% Nonidet P-40) to a final volume of 0·05 ml and incubated at 37°C for 10 min. Subsequently, 0·05 ml of reaction mixture II (20 nmol [$^3$H]-dGTP, 100 ct/min/pmol$^{-1}$; and 3·33 $\mu$g poly(rC)-oligo(dG)) was added and further incubated for 60 min at 37°C. The acid-precipitable radioactivity was collected on a glass-fibre filter, dried and counted in a Nuclear-Chicago, IPSCO/300 liquid-scintillation counter. Murine leukaemia virus, Moloney strain, currently under study in the laboratory of one of us (P.K.Y.W.) was used as a positive control.

The results of assays for RDDP activity in CMT-14B were as follows: The supernatant from cell cultures treated with IUdR and dexamethasone had radioactivity counts above background of 1293 ct/min at passage 18, and 1034 at passage 39. These counts were regarded as positive. The radioactivity counts in untreated control cell cultures were 141 and 249 for passages 18 and 39, respectively, and consequently were interpreted as negative.

(2) *Determination of buoyant density.*—Since the treated cell cultures revealed RDDP activity, it was decided to determine the buoyant density at which the activity banded. Cell cultures at passage 39 were incubated with IUdR and dexamethasone as described above, after which the medium was replaced with a fresh medium containing 33 $\mu$Ci/ml [$^3$H] and further incubated at 37°C for 24 h. Subsequently, the fluid was removed, the cultures washed with phosphate-buffered saline and incubated in a fresh medium for an additional 24 h. The supernatant was then collected from several flasks, pooled and used in the tests. The fractions obtained following centrifugation at 284,000 *g* for 15 h in a SW 40 rotor were precipitated in cold 10% TCA for 20 min. The precipitate was then collected on 0·45 millipore filter, washed, dried and radioactivity counted in a liquid-scintillation counter.

The assays for the buoyant density in treated cultures revealed peaks of radioactivity in a region corresponding to densities of 1·16 to 1·18 g/cm$^3$ (Fig. 1). This range of the buoyant density is characteristic of the known oncornaviruses.

FIG. 1.—Buoyant density in sucrose gradient of oncornavirus-like particles present in culture medium of CMT-14B cell culture treated with IUdR and dexamethasone.

FIG. 2.—Virus-like particle budding in cytoplasmic cisterna of CMT-14B cell. × 150,000.

FIG. 3.—Mature virus-like particle with dense, ring-like, centrally placed nucleoid in cytoplasmic vacuole. × 140,000.

FIG. 4.—Mature virus-like particle with dense nucleoid in intercellular space. × 140,000.

(3) *Electron microscopy*.—The morphology of virus-like particles was studied in sectioned preparations of pellets obtained from culture fluid, and in sections of intact cells at passages 18 and 39. The pellets were obtained from culture fluid after an initial low-speed clarification and subsequent centrifugation at 113,000 *g* for 3·5 h. They were then fixed in a 2% aqueous solution of glutaraldehyde, post-fixed in a 2% solution of osmium tetroxide, dehydrated in ethanol of ascending grades and embedded in Epon. The sections of pelleted material were stained with uranyl acetate and lead citrate according to the method of Venable & Coggeshall (1965). The presence of virus particles was also studied in sections of intact CMT-14B cells. For that purpose the cell cultures were harvested, fixed in 2% solution of glutaraldehyde, post-fixed in 2% osmium tetroxide and processed as described above. Pellets obtained from control culture cells and supernatant fluid were processed according to the same technique.

Studies of sectioned preparations of treated CMT-14B cells revealed oncornavirus-like particles. The number of the particles, however, was small, not exceeding one particle in ~20 cell profiles. The particles budding in cytoplasmic vesicles (Fig. 2) or from the plasma membrane had an average diameter of 100 nm. A single, shell-like layer of densely staining material evidently representing the nucleoid was juxtaposed with the inner leaflet of the delimiting membrane. In mature particles the nucleoid had a closely apposed inner delimiting membrane and was positioned centrally (Figs 3 and 4). Type A particles were not observed in CMT-14B cells.

Examination of the sectioned pellet obtained from the supernatant fluid of treated cultures revealed the presence of a small number of oncornavirus-like particles ranging in diameter from 100 to 130 nm. Their outer membrane had a typical bilaminar structure and was 7 to 8 nm thick. The nucleoid, measuring 50 to 75 nm, was condensed and concentrically placed. No virus particles were detected in control culture fluid or cells.

Although the number of CMT-14B virus particles observed was small, a preliminary analysis of their morphology in comparison with that of the known oncornaviruses is warranted. Mature CMT-14B particles, by central location of their nucleoid and a closely apposed inner delimiting membrane, differ from type-B murine mammary-tumour virus (MuMTV) (Sarkar & Moore, 1972) and Mason–Pfizer monkey virus (M-PMV) (Sarkar and Moore, 1972; Kramarsky *et al.*, 1971) and resemble type-C viruses, including

R-35 rat mammary-tumour virus (Chopra *et al.*, 1970). Furthermore, CMT-14B particles lack the surface "spikes" in common with type-C and M-PMV viruses. However, the structure and positioning of the nucleoid of the budding CMT-14B particles appears to be significantly different from that of the type-B and type-C viruses, being single-shelled and juxtaposed with the inner leaflet of the delimiting membrane. This characteristic resembles the structure of the nucleoid of budding forms of bovine leukaemia virus (BLV) (Calafat & Ressang, 1977). In contrast to BLV, however, no particles free in the cytoplasm were observed in CMT-14B cells. The nucleoids of mature CMT-14B and BLV particles also appear different.

These studies tend to indicate that CMT-14B virus-like particles may represent an endogenous oncornavirus chemically activated in CMT-14B cell cultures. This observation is supported by the negative results obtained from untreated cultures. Consequently, the possibility of contamination of cultures by an unrelated oncornavirus, *e.g.*, from serum in the culture medium, can be discounted. Furthermore, no RNA tumour viruses have ever been maintained or propagated in our (A.M.W.) laboratory.

We wish to thank Drs D. W. Macy and C. W. Smith of the Department of Clinical Veterinary Medicine, University of Illinois for a generous supply of tumour tissue; Drs D. V. R. Reddy and H. T. Hsu of the School of Life Sciences, University of Illinois for technical assistance and Mr P. J. Puca for photography.

### REFERENCES

CALAFAT, J. & RESSANG, A. A. (1977) Morphogenesis of bovine leukemia virus. *Virology*, **80**, 42.

CALAFAT, J., WEIJER, K. & DAAMS, H. (1977) Feline malignant mammary tumours. III. Presence of C-particles and intracisternal A-particles and their relationships with feline leukemia virus antigens and RD-114 virus antigens. *Int. J. Cancer*, **20**, 759.

CHOPRA, H. C. & MASON, M. M. (1970) A new virus in a spontaneous mammary tumour of a rhesus monkey. *Cancer Res.*, **30**, 2081.

CHOPRA, H. C., BOGDEN, A. E., ZELLJADT, I. & JENSEN, E. M. (1970) Virus particles in transplantable rat mammary tumour of spontaneous origin. *Eur. J. Cancer*, **6**, 287.

FELDMAN, D. G. & GROSS, L. (1971) Electron microscopic study of spontaneous mammary carcinomas in cats and dogs: virus-like particles in cat mammary carcinomas. *Cancer Res.*, **31**, 1261.

FINE, D. L., PLOWMAN, J. K., KELLY, S. P., ARTHUR, L. O. & HILLMAN, E. A. (1974) Enhanced production of mouse mammary tumour virus in dexamethasone treated, 5-iododeoxyuridine-stimulated mammary tumour cell cultures. *J. Natl. Cancer Inst.*, **52**, 975.

KRAMARSKY, B., SARKAR, H. H. & MOORE, D. H. (1971) Ultrastructural comparison of a virus from a rhesus-monkey mammary carcinoma with four oncogenic RNA viruses. *Proc. Natl. Acad. Sc. U.S.A.*, **68**, 1603.

SARKAR, H. H. & MOORE, D. H. (1972) Electron microscopy in mammary cancer research. *J. Natl. Cancer Inst.*, **48**, 1051.

VENABLE, J. H. & COGGESHALL, R. (1965) A simplified lead citrate stain for use in electron microscopy. *J. Cell Biol.*, **25**, 407.